# Audience Responses to Online Public Shaming in Online Environments: Mixed Methods Study

**DOI:** 10.2196/67923

**Published:** 2025-07-23

**Authors:** Annie T Chen, Lea H Dunn, Wei Fan, Nidhi Agrawal

**Affiliations:** 1 Department of Biomedical Informatics and Medical Education University of Washington School of Medicine Seattle, WA United States; 2 Foster School of Business University of Washington Seattle, WA United States; 3 Brooks Running Seattle, WA United States

**Keywords:** online public shaming, social media, mental health, social acceptability, emotions, social norms, COVID-19

## Abstract

**Background:**

Incidents of online public shaming can have devastating consequences for those who are shamed, but how those who witness shaming behaviors react is unclear. When considering online public shaming, it is crucial to be aware of the context in which it occurs. Implicit norms that govern these contexts and evoke emotions can influence what is deemed as acceptable behavior when witnessing public shaming. However, previous work has not examined the role that perceived social acceptability of the shaming content and emotional arousal may have in explaining social media behavior after witnessing online shaming incidents.

**Objective:**

We posed three research questions to explore and characterize people’s reactions to witnessing online public shaming: (1) Does perceived social acceptability predict subsequent engagement with tweets; (2) Are participants’ emotional reactions to tweets associated with self-assessed likelihood to engage in certain behaviors; and (3) What do participants’ explanations of their reactions to tweets illustrate about their views about appropriate online behavior?

**Methods:**

We conducted a between-subjects experimental design in which participants saw 1 of 4 tweets: shaming of a couple holding a wedding that became a super-spreader event (shaming condition) and 3 control tweets: 1 expressing positive sentiment about a wedding during the COVID-19 pandemic(wedding condition), a public service announcement providing information about obtaining COVID-19–related information (COVID-19 condition); and a tweet expressing neutral sentiment (control condition). To answer the first 2 questions, we constructed regression models with replying or commenting, sharing, and liking as the dependent variables and factors including demographic characteristics, social media behaviors, tweet type, perception of social acceptability, and emotions as the independent variables. To characterize how participants interpreted the online act, we performed an inductive qualitative analysis of open-ended responses explaining participants’ reactions to the tweet they saw, and then compared the prominence of the themes across the conditions using ANOVAs.

**Results:**

We invited 800 participants, and 742 participants completed the entire study. Regarding the first research question, the perception of a tweet as socially acceptable increased all forms of social engagement, with increased liking, sharing, and commenting. Regarding emotions, positive emotions were associated with a greater tendency to engage in all 3 behaviors, whereas fear, shame, and anger involved more interactions between the tweet type, emotion, and the subsequent behavior (liking, sharing, or commenting). Last, qualitative analysis of the open-ended responses yielded a model of media interpretation and engagement in online environments comprising 5 factors: environment, sense-making and assessment, message, tone, and other, with substantial differences in the themes across the tweet types.

**Conclusions:**

This study augments our understanding of people’s responses to online public shaming, including the diverse factors that may affect interpretations and subsequent action. Considering these factors in platform design can mitigate negative consequences.

## Introduction

### Background

Online shaming is a form of public shaming in which individuals are harassed, mocked, or bullied by other individuals. There can be devastating consequences of online public shaming, such as losing ones’ employment, facing public criticism, negative publicity, and even suicide [1-4]. Public shaming occurs when a person is characterized in a morally stigmatizing way for personal characteristics or behaviors that may be contrary to a standard, social norm, or moral code [1,5]. Shaming acts enforce societal norms by humiliating those who violate them, which acts as a form of punishing transgressors and deterring others from performing the same behaviors [6]. The COVID-19 pandemic gave rise to situations of shaming, for example, when people did not follow lockdown rules, and shaming language, such as “covidiot,” took on a newfound prominence [7]. Situations can have devastating consequences, such as in the case of Wilson Gavin, whose opposition to aspects of LGBTQ (lesbian, gay, bisexual, transgender, queer) culture resulted in an internet pile-on and online shaming, ultimately resulting in his taking his own life [4]. However, the COVID-19 pandemic is but one situation in which shaming could occur; negative sentiment regarding other health issues, such as obesity have also been observed in social media [8].

There is substantial interest concerning how online public shaming develops [3,9], the factors that predict it [10], and its justifiability [1,11]. Online public shaming can negatively impact those involved, at times with severe consequences for mental health [1-3]. However, there has been limited attention to how the public perceives it [4], and to our knowledge, how it affects those who witness it and their subsequent reactions.

Understanding the mental health implications of online public shaming is paramount to avoid escalations of sensitive situations; moreover, there is also substantial concern about the effect of social media use more broadly on well-being, including a need for more attention to behavioral assessment (rather than self-report) and effect heterogeneity [12], and the need to consider smaller effects that might contribute to larger implications for well-being [13]. Thus, in this study, we conducted an experiment to better understand how people might react to public shaming in digital spaces and the underlying factors that shape online behavioral responses to shaming content. However, to do so, we first consider literature on norms, emotion, and acceptable behavior on social media.

### Implicit Norms, Emotions, and Engagement on Social Media

When we consider online public shaming, it is important to consider the context in which it occurs. People use social media platforms to suit different purposes. For example, the need to belong and the need for self-representation have been argued as 2 main motivations for Facebook use [14]. Depending on the medium, people engage in self-creation and self-representation differently [15,16]. People may also have other motivations for engaging with social media, including enjoyment, self-efficacy, learning, personal gain, altruism, empathy, community interest, and social engagement [17].

Social norms are defined as a set of normative expectations around the ways individuals should and should not behave. Collective norms, or what the aggregate group would believe is socially acceptable [18], are not the only norms that govern behavior. Perceived social norms also influence people’s actions, but also how people respond to perceived violations of norms. Public behavior is often influenced by perceived injunctive norms, or what an individual believes their group would find socially acceptable [18]. Observational research also suggests that there are implicit norms on social media, and these can differ depending on the platform [19-22]. Differing norms on platforms can be an artifact of available features on the apps, how people interact on them, and whether there was a previous relationship. Facebook, Instagram, and Snapchat are mostly used for sharing positive emotions, and Twitter (subsequently rebranded X) and Messenger are often used for sharing negative emotions [19]. Sharing on Facebook is a means of garnering social acceptance, but not everything should be shared, and seeking attention is deemed inappropriate [22]. On platforms involving family, such as Facebook, people may find posting extremely personal information inappropriate, and “you’d want to keep things pretty tame” [17].

### Social Acceptability and Emotion in Social Media

Social acceptability refers to the degree to which a behavior, belief, or idea is considered acceptable or approved of by a social group. To be socially acceptable, it must align with the prevailing norms and values within a community. But what is perceived to be socially acceptable can be determined by a variety of factors, including the implicit norms of the social platform and the individual’s perceived values, among others. Social acceptability perceptions can shape what kinds of content are shared, where unhealthy or stigmatized behavior could be perceived as less socially acceptable and thus lead to lower sharing [23]. Unacceptable behavior can include rude or offensive postings, coarse language, too much information, and breaching others’ privacy [20]. Aside from norms of the platforms themselves, there may also be aspects of social media content that affect engagement. For example, sensory and visual features might lead to liking, rational and interactive content to commenting, and sensory, visual, and rational content to sharing [24].

Emotional arousal tends to increase engagement, and emotional valence acts as a facilitator [25]. For example, emotionally charged tweets tend to be retweeted more often than neutral ones [26,27]. In marketing research, more positively and negatively framed comments increase engagement on Facebook [28], emotion-focused advertisements tend to be more shared [29], and content evoking high arousal generates greater sharing intention [30].

However, emotions can be highly differentiated. For example, previous work on crisis and event communications has observed that not all emotions elicited the same subsequent behavior [27,31]. Immediately following a terror attack, fear was negatively associated with retweeting, and retweet behavior varied with respect to specific types of negative affect, including anxiety, depression, and contempt [31]. According to cognitive appraisal theory, a person experiences a pattern of emotions systematically along certain dimensions based on their appraisal of an event [32]. The complexity of emotional responses that can arise in online public shaming call for a more nuanced analysis of the emotional responses and how they might affect subsequent behavior.

### Research Questions

Public shaming in online spaces may be more common as social media can reduce moral sensitivity, and lower perceived moral sensitivity can give rise to a greater tendency to participate in online shaming [33]. In recent years, public shaming on Twitter has been a common phenomenon [2].

A fundamental underpinning of shaming is rooted in the understanding that all involved share a collective moral standard [6]. This moral standard creates expectations for “appropriate” behavior and puts the onus on others to uphold this social norm. When one witnesses online public shaming, they are faced with an ethical dilemma of whether to take part or support the action, which may cause harm to the person being shamed. In reality, people may differ in the extent to which they agree with a norm, and situations, such as the COVID-19 pandemic, gave rise to uncertainty concerning norms for behaviors such as social distancing and mask wearing. Previous work has reported that tweets expressing the expected emotional valence in a given situation are more likely to be retweeted [34], but the expected emotional valence for shaming content may be in question where the social norm itself is in flux.

As such, this research seeks to explore the interplay of perceived social acceptability and elicited emotion in social media, particularly in situations where the content portrays controversial behaviors. We focus on the witness to online public shaming, rather than the direct consequences of it for those subject to or engaged in it, to better understand how it affects all parties. The COVID-19 pandemic was a public health context where a behavior choice (eg, wearing a mask, or holding a social event such as a wedding) could be linked to public health consequences (ie, spreading the COVID-19 infection). Due to the change in guidelines, variation in risk tolerances, and trade-offs, there were incidents of shaming related to social distancing and mask wearing both offline and online, in mass media and on social media [35,36].

We conducted an experiment in which study participants were exposed to 1 of 4 tweets. One tweet shamed the featured persons for a behavior that had public health consequences (ie, holding a super-spreader wedding), and 3 tweets served as controls for different aspects of the shaming tweet. Participants were asked to answer multiple-choice and open-ended questions about their reaction to the tweet and their likelihood to interact with it in terms of liking, commenting or replying, and sharing. These are common forms of engagement on social media; thus, understanding the motivations and factors that influence each can be valuable. We constructed a set of regression models with these behaviors as the dependent variables to explore how social acceptability and emotion may vary across the shaming and other conditions through the following research questions (RQs):

- Does the perceived social acceptability of a tweet predict the likelihood to engage with it in terms of liking, commenting or replying, or sharing? (RQ 1)
- Are participants’ emotional reactions to the tweet associated with the likelihood to engage in these behaviors? (RQ 2)

Although the regression models will enable us to investigate the influence of social acceptability and emotion on subsequent behavior in the different conditions, how a person views shaming incidents amidst the uncertainty of the COVID-19 pandemic and the online environment is unclear. Thus, in our last RQ, we performed a qualitative analysis of open-ended responses to questions about participants’ reactions:

- What do participants’ explanations for their responses to the tweets illustrate about how they interpret and decide what is appropriate online behavior? (RQ 3)

## Methods

### Study Design

We conducted an experimental study in which participants were exposed to 1 of 4 tweets. We performed analysis of the data using a mixed methods approach, in which researchers collect data and analyzed it according to both quantitative and qualitative approaches [37,38]. We first used regression models to investigate factors that may affect audience behavior after viewing tweets; we complemented this with a qualitative analysis that “unpacks” the phenomenon further, and then compared the prominence of themes across the tweet types. In this context, the integration of quantitative and qualitative methods served to facilitate triangulation and increase the completeness of our characterization of the phenomenon of online public shaming [37].

### Data Collection

In this study, we used Prolific Academic (Prolific Academic Ltd), a platform for online behavioral research, to recruit participants. Prolific has been reported as having higher data quality than other currently available platforms [39,40]. To conduct a study using the platform, researchers post study descriptions. Persons registered with Prolific to participate in research read the study descriptions and participate if they are interested. Participating multiple times is prevented through the use of unique IDs for each participant, while also preserving participants’ privacy, because participants’ identifiable information is not disclosed to researchers. Data were collected on Qualtrics (Qualtrics International Inc), a commonly used survey platform [41]. Once their participation was complete, participants were compensated for their time. For more details on consent and compensation procedures, see the Ethical Considerations section.

Data were collected over 2 days in March and April 2022. On the first day, participants completed part 1, answering questions about participants’ demographic characteristics, social media use, perceptions of the COVID-19 pandemic situation, and willingness to engage in preventive behaviors. Twenty-four hours later, participants were contacted to complete part 2. Participants were randomly assigned to view 1 of 4 tweets and asked about their reactions. The reason for separating the 2 days of data collection was to avoid participants’ answers concerning their reactions to the tweet being influenced by their answers about their social media use and behaviors. The stimuli were pretested to ensure that the contents of the tweets aligned with what we were testing.

During part 1, we collected information about participants’ demographic characteristics (age, gender, race or ethnicity, whether they were a native English speaker, and political ideology). We also asked participants about their social media use, including frequency of use of different social media (eg, Facebook, Instagram, TikTok, and YouTube), and their perception of the risk of not wearing a mask indoors.

**Figure 1:**
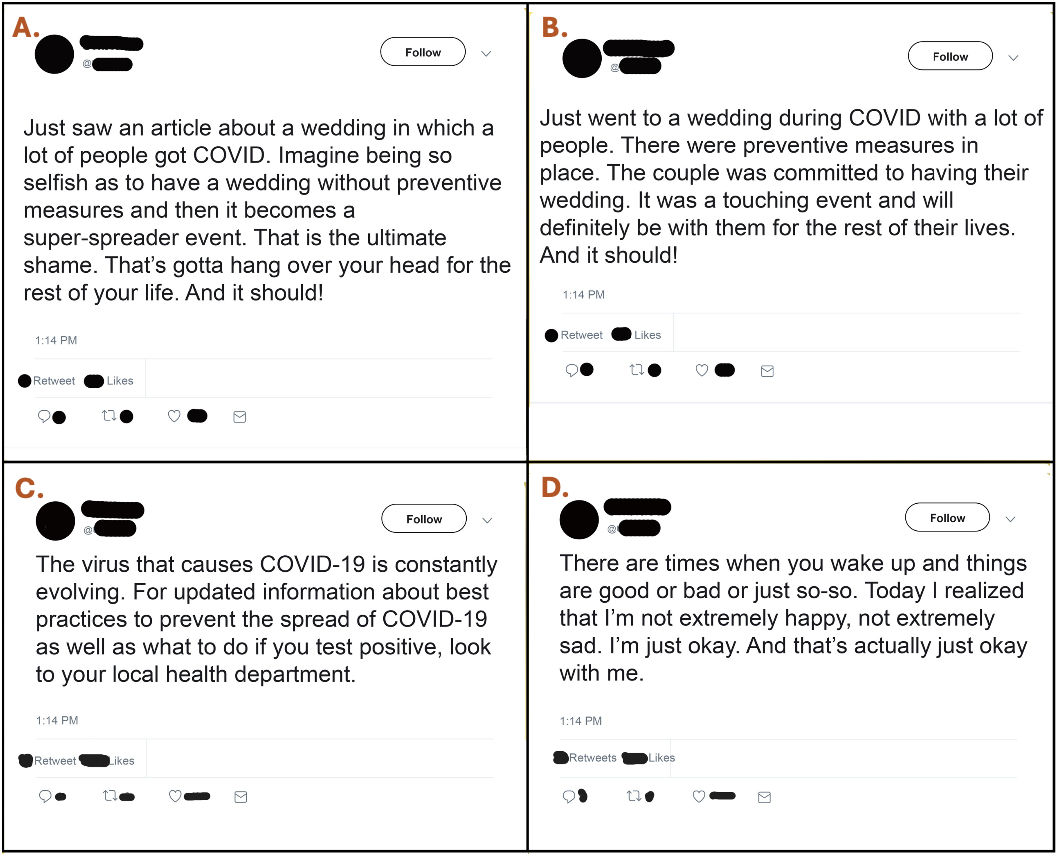
Tweet stimuli: (A) shaming tweet; (B) wedding tweet; (C) COVID-19 informational tweet; and (D) control tweet.

In part 2, we developed and pretested 4 tweets with different tenors (hereafter called “types”) and randomly allocated each participant to view 1 of the 4 tweets and answer questions about the tweet (Figure 1). In the first tweet, the poster shames a couple who held a wedding that became a super-spreader event (*shaming condition*).

The remaining 3 tweets served as different types of controls. One tweet expressed positive sentiment about a wedding during the COVID-19 pandemic, and clearly states that precautions were in place to keep everyone safe, intended to control for the perception that those who held the wedding were irresponsible (*wedding condition*). Another tweet was framed as a public service announcement, providing information about how to obtain COVID 19-related information, thus controlling for any inherent negative association of the COVID-19 pandemic (*COVID-19 condition*). Finally, we developed a personal tweet expressing neutral sentiment, without the potential impacts of either of these 2 aspects (*control condition*).

The 4 tweet conditions were pretested to ensure that they successfully manipulated the emotions and responses intended by the content. The pretest concluded that the shaming tweet was seen as significantly more intended to shame, judge someone, and vent about something (*P*<.001 for all comparisons), the wedding tweet was more intended to celebrate someone (*P*<.001 for all comparisons), the control tweet was intended to share an experience (*P*<.001 for all comparisons), and the COVID-19 pandemic tweet was intended to share information (*P*<.001 for all comparisons). We pretested the tweets as a means of determining the correct message, as well as avoiding a manipulation check that might draw attention toward the intention of the tweets and away from how the participant might respond to the tweet itself.

After viewing one of the 4 tweets, all participants completed the same questionnaire concerning their reactions, including the emotions experienced, their likelihood of commenting or replying, sharing, and liking the tweet, and their perception of the social acceptability of the tweet. We also asked participants an open-ended question concerning their thoughts about the post, and to explain the reasons for the social acceptability rating that they gave. Questions that were part of a set (eg, types of social media used, emotions) were automatically randomized in presentation for each participant. In this study, we focused on participants’ perceptions of social acceptability of the post, the emotions experienced, but all the questions for the survey are provided in Multimedia Appendix 1.

### Data Analysis

#### Participant Characteristics

800 participants completed part 1, and 742 participants returned to complete the part 2 (completion rate: 92.75%). Data analysis was conducted with the participants who completed part 2 (N=742). We calculated aggregate statistics for the sample (on age, gender, political ideology, and social media use and behavior) and confirmed that no systematic differences in demographics existed between the conditions using a series of ANOVAs.

The study variables are summarized in Table 1. The cognitive appraisal theory of emotion suggests that different emotions are distinctly different in both their appraisal construction and action motivation [32]. For example, shame is a negative emotion with internal attributions of blame, meaning the self is the cause of the issue. Shame is an emotion that leads to withdrawal or avoidance as a way of protecting the self [42]. Fear is defined as low in personal control and high uncertainty [43] and often results in desire for greater information to reduce uncertainty and help decide between avoidance or approach, and anger is defined by other responsibility which typically motivates someone to act against the source of the anger. On the basis of these theoretical underpinnings, we expected that the different negative emotions would act differently from each other and from positive emotions.

As people’s reactions to social media content can be complex, especially in crisis situations [44], we sought to capture variation in emotion through 10 items selected from the Positive and Negative Affect Schedule (PANAS) scale [45]. We derived composite indicators for social acceptability based on the 3 individual items (α=.89), and for 3 emotion families based on the 10-item PANAS by averaging the items for positive, fear, and anger emotions. We used shame as a single item. Multimedia Appendix 2 provides details concerning the derivation of composite indicators.

**Table 1:** Study variables.

Survey part and variable		Description	Measure
**Survey part 1**			
	Demographics	Age	Interval or ratio
	Demographics	Gender, race or ethnicity, native English speaker, and political ideology	Multiple choice political ideology was measured using 3 Likert-type items (1=strongly liberal and 7=strongly conservative) [46]
	Routine social media use	Frequency of use of different social media (Facebook, Instagram, TikTok, YouTube, among others)	Likert-type items (0=no use; 6=more than 9 times a day)
**Survey part 2**			
	Tweet type	Condition that the participant was assigned to	Shaming, wedding, COVID-19, neutral
	Emotions	10 items from the PANAS^a^ [45]	Likert-type items (1=not at all; 9=a lot)
	Behaviors	Likelihood of commenting or replying, sharing, and liking the tweet	Likert-type items (1=extremely unlikely; 5=extremely likely)
	Social acceptability of the tweet	Social acceptability index	Likert-type items (1=not at all X; 5=very X), where X=socially responsible, acceptable, or appropriate

^a^PANAS: Positive and Negative Affect Schedule.

To explore potential for multicollinearity, we calculated tolerance and variance inflation factor [47]. Collinearity statistics revealed no significant issues apart from multicollinearity in the interaction terms, which is to be expected and not an issue in this analytic method [48,49].

#### RQs 1 and 2: Social Acceptability and Emotion

We constructed linear regression models to explore the first and second RQs. Each model included a common set of control variables (personal characteristics and social media use); the manipulated condition, tweet type; and one unique component. Whereas RQ1 sought to examine the effect of perceived social acceptability, RQ2 aimed to understand the effect of emotional reactions on social media message engagement (ie, likelihood to comment or reply, share or retweet, and like) after viewing the tweet (Table 2).

**Table 2:** Regression model overview for control and research questions (RQs).

Independent variables	Control	RQ1	RQ2
Personal characteristics	✓	✓	✓
Social media use	✓	✓	✓
Tweet type	✓	✓	✓
Social acceptability		✓	
Emotional reactions			✓

#### RQ 3: Analyzing Audience Responses to Online Shaming Behavior

Although extant knowledge exists concerning online public shaming, gaps exist concerning how viewers might appraise a situation and, in response, react to it. To address this gap, we performed an inductive qualitative analysis, an appropriate analysis method when there is not enough knowledge about a phenomenon, or that knowledge is fragmented [50]. We performed an inductive qualitative analysis of the responses to 2 open-ended questions concerning participants’ perceptions of the tweets and the reason for the social acceptability rating they gave. This inductive process begins with a close reading of the text, followed by identification of relevant segments, code assignment, reduction of codes by considering overlap and redundancy, and creation of a schema including the most salient themes [51].

Qualitative coding involved 2 main phases. In the first phase, 2 co-authors independently identified themes in a random sample of responses from each of the 4 conditions, blinded to the conditions. The coders discussed their codes, and a third author reviewed these codes and provided guidance on code refinement on iterative subsets until agreement upon a coding scheme was reached. Interrater reliability, measured by Cohen κ, reached 0.79 and 0.77 on the 2 questions, indicating substantial agreement [52]. To maximally use the available data, we resolved the codes in this initial phase by consensus. In the second phase, the 2 coders then independently coded the remaining tweets, and a final code determination was made on each code by averaging the codes of the 2 coders. We then performed ANOVAs to compare the prominence of each theme across conditions.

### Ethical Considerations

Participants were informed that they were taking part in a study of reactions to social media conducted by the University of Washington, that the study would take place over 2 days, and they would receive compensation for their efforts via Prolific Academic [53]. For completing part 1, participants received US $0.80, and for completing part 2, participants received US $1.20 (a total of US $2.00). With respect to privacy and confidentiality, participants were informed that the data would be collected and securely stored on the Qualtrics survey platform [41], and that only study investigators would have access to these data.

In conducting this research, we sought to protect the privacy and confidentiality of the participants in various ways. First, we used the Prolific platform, which protects participants’ privacy by assigning each participant a unique ID (thus facilitating pseudo-anonymization), not storing data collected in research studies, and restricting researchers from accessing participants’ identifiable information [54]. In addition, in the study design and data analysis, we: (1) did not ask for personal information in the open-ended questions; (2) reported data in aggregate where possible; and (3) ensured that the sample quotations are deidentified. Participants gave consent for the study procedures before their participation, and all study procedures were approved by the University of Washington Human Subjects Division (STUDY00010534). To ensure rigor in the execution and reporting of this study, we employed the Checklist for Reporting Results of Internet E-Surveys (CHERRIES) [55] (Multimedia Appendix 3). Where appropriate, we have also explained our rationale for our data quality procedures. For example, as duplicate IP addresses are not necessarily an indicator of fraudulent data [56,57], we did not exclude responses based on IP address.

## Results

### Participant Characteristics

The average age of the sample was 37 (SD 13; range 18-79) years, and of the 742 participants, 470 (63.3%) were women participants (Table 3). Most participants in the sample were White (598/742, 80.6%) and non-Hispanic (683/742, 92%); almost all were native English speakers (723/742, 94.7%). While the sample varied in terms of political ideology, overall, it was left-leaning (mean score 3.1, SD 1.7).

**Table 3:** Participant characteristics (N=742).

Characteristics		Participant statistics
Age (y), mean (SD)		37 (13)
**Gender, n (%)**		
	Men	255 (34.4)
	Women	470 (63.3)
	Nonbinary	16 (2.2)
**Race, n (%)**		
	Asian	56 (7.5)
	Black	47 (6.3)
	Native American	5 (0.7)
	White	598 (80.6)
	Other	29 (3.9)
	Prefer not to answer	6 (0.8)
**Ethnicity, n (%)**		
	Hispanic	57 (7.7)
	Non-Hispanic	683 (92)
**Native English speaker, n (%)**		
	No	18 (2.4)
	Yes	723 (97.4)
Political ideology score^a^, mean (SD)		3.08 (1.67)

^a^1=strongly liberal and 7=strongly conservative.

Almost all study participants engaged in some form of social media use (Table 4). Among the 742 participants, YouTube was the most common form of social media to use, with 717 (96.6%) reporting at least some use, but Facebook, Instagram, Reddit, and Twitter were also used by 498 (67.2%) of the sample or more. Overall, participants self-reported that they were unlikely to share or retweet social media content (mean 2.31, SD 1.21); somewhat more likely, but still not likely, to comment or reply (mean 2.66, SD 1.15); and between neutral and likely to like a post on social media (mean 3.73, SD 1.15).

**Table 4:** Participants’ social media use (N=742).

Social media	Yes^a^, n (%)	No, n (%)
Twitter	498 (67.2)	243 (32.8)
Facebook	608 (82.1)	133 (17.9)
Instagram	565 (76.1)	177 (23.9)
TikTok	404 (54.5)	337 (45.5)
Snapchat	317 (42.7)	425 (57.3)
YouTube	717 (96.6)	25 (3.4)
Pinterest	405 (54.7)	336 (45.3)
Reddit	564 (76)	178 (24)

^a^“Yes” denotes any response other than 0 (ie, “N/A” or “I do not use”).

### RQ 1: Does the Perceived Social Acceptability of a Tweet Predict Behavior After Viewing the Tweet?

#### Overview

In our first RQ, we sought to understand how social acceptability of a tweet might predict subsequent engagement behaviors. We constructed 3 regression models with the likelihood to like the tweet, share the tweet, and comment on the tweet as the dependent variables (Table 5). Personal characteristics, social media use, tweet type, perceived social acceptability, and interaction terms involving each condition and social acceptability were the predictors.

**Table 5:** Research question 1: regression coefficients that model the effect of perceived social acceptability on social media engagement of liking, sharing or retweeting, and commenting or replying.

Variables		Like^a^ (regression coefficient, b)	Share or retweet^b^ (regression coefficient, b)	Comment or reply^c^ (regression coefficient, b)
Intercept		−1.701^d^	−0.620	−0.563
**Personal characteristics**				
	Age (y)	0.006	0.001	0.012^d^
	Gender D1^e^ (men vs women participants)	0.031	−0.116	−0.258^f^
	Gender D2^g^ (men vs nonbinary participants)	−0.056	0.258	−0.032
	Political ideology	−0.001	0.039	0.078^f^
**Social media use**				
	Twitter	0.061^h^	0.047^i^	0.029
	Facebook	0.044	0	0.076^d^
	Instagram	−0.012	0.019	0.015
	TikTok	0.077^f^	0.069^f^	0.048^i^
	Snapchat	0.046	−0.015	−0.010
	YouTube	0.021	0.037	0.016
	Pinterest	0.147^f^	0.198^d^	0.237^d^
	Reddit	−0.052^i^	−0.029	−0.12
	C1^j^	0.613	0.613	0.703^i^
	C2^k^	0.900^i^	0.803^h^	0.322
	C3^l^	0.375	0.003	0.882^i^
**Social acceptability**				
	Social acceptability index	1.055^d^	0.515^d^	0.365^d^
	C1×social acceptability	−0.228^i^	−0.167	−0.184^i^
	C2×social acceptability	−0.306^h^	−0.296^f^	−0.106
	C3×social acceptability	−0.283^h^	0.013	−0.274^h^

^a^*R*=0.643; *R*^2^=0.414; SE 1.176.

^b^*R*=0.492; *R*^2^=0.242; SE 0.995.

^c^*R*=0.424; *R*^2^=0.180; SE 0.981.

^d^*P*<.001.

^e^D1: dummy coded gender (men vs women).

^f^*P*<.01.

^g^D2: dummy coded gender (men vs nonbinary).

^h^*P*<.05.

^i^*P*<.10.

^j^C1: condition *shaming* versus *control*.

^k^C2: condition *wedding* versus *control*.

^l^C3: condition *COVID-19* versus *control*.

#### Demographic Characteristics

The results suggested that certain demographics and previous social media behavior influence the likelihood of engaging in subsequent social media behavior. No demographic variables predicted the likelihood of sharing or retweeting a post. For commenting, older participants were more likely to comment (b=0.012; *P*<.001), men were more likely to comment as compared to women (b=−0.258; *P*=.005), and a right-leaning political ideology was associated with increased likelihood to comment (b=0.078; *P*=.001).

Examining the correlation between age and social media platform use, older people were more likely to have a higher frequency of Facebook use (*r*=142; *P*<.001). Given that this platform encourages more commenting-style engagement, it perhaps makes sense that older participants would be more likely to engage in commenting behavior. Age was also highly positively correlated with increased conservatism (*r*=.218; *P*<.001), which may also explain why more conservative participants may have been more likely to comment.

#### Social Media Use

When examining previous social media use, it appeared that engaging on certain social media platforms influenced subsequent social media post responses. Increased Twitter use influenced the likelihood of liking (b=0.061; *P*=.03) and sharing (b=0.047; *P*=.05), but not commenting. Higher frequency of Facebook use influenced the likelihood of commenting (b=0.076; *P*<.001), but not liking or sharing. Use of TikTok and Pinterest were both associated with increased likelihood of engaging in all 3 behaviors, and use of Reddit decreased the likelihood of liking (b=−0.052; *P*=.06).

#### Social Acceptability and Tweet Type

When tweets were seen as socially acceptable, people were more likely to engage with them in terms of all 3 behaviors (liking: b=1.055, *P*<.001; sharing: b=0.515, *P*<.001; and commenting: b=0.365, *P*<.001). However, there were also differences in intention to engage in behaviors based on the tweet seen. The positive tweet about a wedding during the COVID-19 pandemic was more likely to be liked and shared as compared to the control, and the shaming and the COVID-19 pandemic informational tweets were both more likely to be commented on or replied to than the control tweet. These results suggest that posts that could be controversial or polarizing, such as posts shaming nonadherence to preventive measures or the topic of the COVID-19 pandemic more generally, were more likely to be engaged with in terms of commenting.

**Figure 2:**
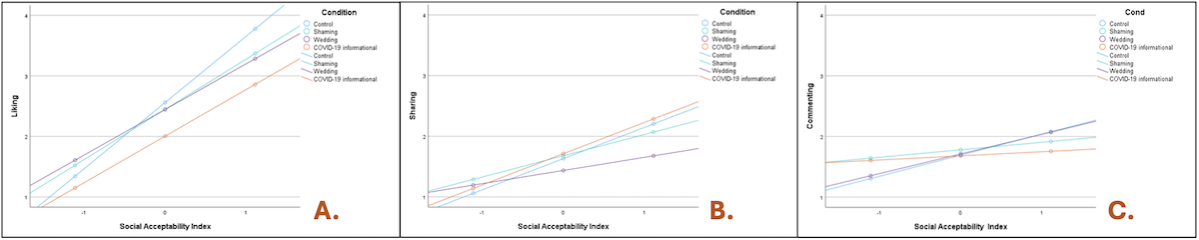
Interaction plots depicting the relationship between perceptions of social acceptability of a tweet and likelihood of social media engagement by type: (A) social acceptability versus liking; (B) social acceptability versus sharing; and (C) social acceptability versus commenting. Blue denotes control, green denotes shaming, purple denotes wedding, and orange denotes COVID-19 informational tweet.

The relationship between perception of the social acceptability of a tweet and likelihood of engagement also varied by tweet type for the behaviors, as illustrated through interaction plots (Figure 2). As social acceptability increased, people were more likely to engage in liking (Figure 2A) for the control (blue) tweet as opposed to the other 3, and in commenting for the control (blue) and wedding (purple) tweets (Figure 2C). However, with respect to sharing, propensity to share increased with social acceptability, for both the control (blue) and the COVID-19 pandemic (orange) tweets, as compared to the shaming and wedding tweets (Figure 2B).

Our main focus was to understand how individual differences in perceived social acceptability might have an effect on subsequent behaviors. However, one might also wonder about the social acceptability of the tweets more generally. An additional analysis was performed to examine whether different topics were perceived to be more or less socially acceptable. An ANOVA using the tweet conditions as the predictor variable and social acceptability as the dependent variable revealed that tweet type influenced perceived acceptability (*F*_3, 738_=55.41; *P*<.001). The shaming tweet (mean 2.95) was perceived to be less socially acceptable than the control tweet (mean 3.77; *P*<.001), the wedding tweet (mean 3.16; *P*=.04), and the COVID-19 pandemic tweet (mean 4.15; *P*<.001). The COVID-19 pandemic tweet was perceived to be the most socially acceptable (compared to all other tweets, *P*<.001). Moreover, a supplemental analysis also using mediation analysis [58] demonstrated that perceived risk also affected perception of social acceptability (Multimedia Appendix 4 [58]). Thus, the content of the tweets can be seen as differentially socially acceptable, which can, in turn, affect how people behave on social media.

### RQ 2: Are Participants’ Emotional Reactions to the Tweet Associated With Self-Assessed Likelihood to Engage in Certain Behaviors?

In this RQ, we examined the role of emotions in the likelihood to like, share, and comment (Table 6).

**Table 6:** Research question 2: regression coefficients of the effect of emotional reactions on social media engagement of liking, sharing or retweeting, and commenting or replying.

Variables		Like^a^ (regression coefficient, b)	Share or retweet^b^ (regression coefficient, b)	Comment or reply^c^ (regression coefficient, b)
Intercept		1.749^d^	1.124^d^	0.486^e^
**Personal characteristics**				
	Age (y)	−0.003	−0.004	0.009^f^
	Gender D1^g^ (men vs women participants)	0.165	−0.008	−0.196^h^
	Gender D2^i^ (men vs nonbinary participants)	0.006	0.348	0.108
	Political ideology	−0.102^d^	−0.008	0.050^h^
**Social media use**				
	Twitter	0.065^h^	0.039	0.011
	Facebook	0.033	−0.011	0.053^h^
	Instagram	−0.035	0.003	0.007
	TikTok	0.027	0.041^e^	0.027
	Snapchat	0.034	−0.015	−0.006
	YouTube	0.040	0.048^h^	0.022
	Pinterest	0.155^f^	0.195^d^	0.216^d^
	Reddit	−0.044	−0.011	−0.002
**Tweet type**				
	C1^j^	−0.613	−0.675^h^	−0.671^h^
	C2^k^	−0.741^e^	−0.240	−0.396
	C3^l^	−0.527	−0.477	−0.690^h^
**Tweet reactions**				
	Positive emotion	0.342^d^	0.224^d^	0.188^d^
	Ashamed	−0.041	−0.214^e^	−0.173
	Fear	0.238^h^	0.027	0.133
	Anger	−0.391^f^	−0.118	−0.109
	C1×positive	−0.128	−0.075	−0.029
	C2×positive	0.030	−0.114^e^	−0.009
	C3×positive	−0.017	0.070	0.044
	C1×ashamed	0.058	0.201	0.224^e^
	C2×ashamed	0.150	0.277^h^	0.209^e^
	C3×ashamed	−0.044	0.028	−0.008
	C1×fear	−0.292^h^	0.023	−0.155
	C2×fear	−0.293^h^	−0.085	−0.119
	C3×fear	0.075	0.298^f^	0.062
	C1×anger	0.443^f^	0.200^e^	0.253^h^
	C2×anger	0.198	0.060	0.073
	C3×anger	0.080	−0.087	0.150

^a^*R*=0.592; *R*^2^=0.350; SE 1.250.

^b^*R*=0.561; *R*^2^=0.315; SE 0.955.

^c^*R*=0.540; *R*^2^=0.291; SE 0.920.

^d^*P*<.001.

^e^*P*<.10.

^f^*P*<.01.

^g^D1: dummy coded gender (men vs women).

^h^*P*<.05.

^i^D2: dummy coded gender (men vs nonbinary).

^j^C1: condition *shaming* versus *control*.

^k^C2: condition *wedding* versus *control*.

^l^C3: condition *COVID-19* versus *control*.

Positive emotional reactions were more likely to increase social media engagement for liking (b=0.342, *P*<.001), sharing (b=0.224; *P*<.001), and commenting (b=0.188; *P*<.001). When examining how reactions to fear and anger influenced subsequent behavior, fear led to an increase in liking (b=0.238; *P*=.03), while anger led to a decrease in liking (b=−0.391; *P*=.002).

Differences emerged in how tweet type influenced subsequent behavior. Overall, respondents were less likely to indicate that they should share (b=−0.675; *P*=.04) and comment (b=−0.671; *P*=.03) the shaming tweet as compared to the control tweet. Compared to the wedding tweet, the control tweet elicited marginally greater liking (b=−0.741; *P*=.07). The control tweet was also more likely to lead to greater commenting than the COVID-19 pandemic tweet (b=−0.690; *P*=.02).

When experiencing greater fear, participants were less likely to like the shaming (b=−0.292; *P*=.026) and wedding (b=−0.293; *P*=.03) tweets. This seems to suggest that when the content as polarizing and resulted in greater fear—particularly in response to a violation of potential COVID-19–preventive behavior—consistent with the avoidance nature of fear, people were less likely to engage with it. However, when feeling greater fear and the content allowed for sharing of information that could help themselves or others assuage the fear (eg, COVID-19 pandemic–related tweet), there was a greater likelihood of sharing (b=0.298; *P*=.003).

The feeling of shame led to a marginally lower likelihood of sharing content (b=−0.214; *P*=.06). The lower likelihood in sharing of shaming content may have been because participants intended to keep attention away from themselves. This has been shown consistently in work related to how people cope with experiencing shaming [42].

While on its own, feelings of shame led to less engagement, when considering different tweet type, heightened feelings of shame resulted in greater commenting on the shaming tweet (b=0.224; *P*=.06), marginally more commenting on the wedding tweet (b=0.209; *P*=.09), and greater sharing of the wedding tweet (b=0.277; *P*=.03). These results suggest that while the experience of shame may be to draw attention away from the self, such avoidance may only occur when individuals see themselves as responsible for a negative outcome.

Finally, when examining how experiencing anger influenced behaviors depending on tweet content, when the tweet was aimed at public shaming (as compared to the neutral tweet), increased anger led to greater engagement across behaviors, resulting in increased liking (b=0.443; *P*=.001), sharing (b=0.200; *P*=.06), and commenting (b=0.253; *P*=.01).

**Figure 3:**
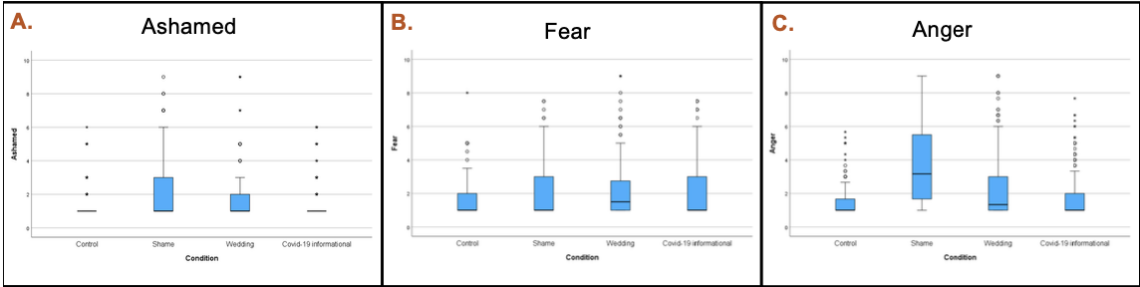
Negative emotions by tweet condition. (A) ashamed; (B) fear; (C) anger. Conditions, left to right: control, shaming, wedding, and COVID-19 informational tweet.

We rendered box plots of the negative emotions by tweet condition to see whether the content differed in the emotions it elicited. On the basis of these boxplots, we found that there was greater variability in the sample with respect to the extent to which the shaming tweet in particular caused reactions of shame and anger (Figure 3).

### RQ 3: What Do Participants’ Explanations for Their Responses to the Tweets Illustrate About How They Interpret and Decide What Is Appropriate Online Behavior?

As described previously, although the regression models afforded a view of the influence of social acceptability and emotion on 3 behaviors, the process by which each individual determined social acceptability was not clear. We performed an inductive qualitative analysis of participants’ open-ended responses to identify the factors that influenced participants’ perceptions of social acceptability on social media.

**Figure 4:**
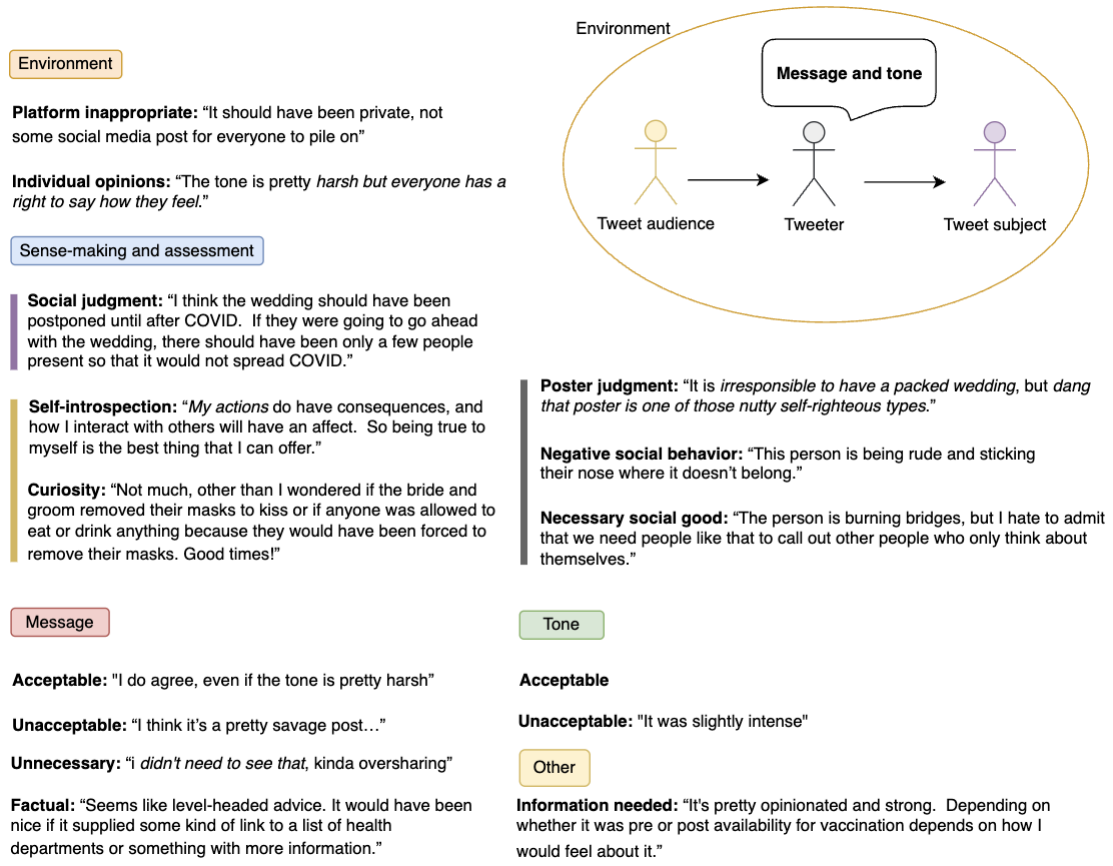
Model of media interpretation and engagement in online environments.

We present an overview of the most prominent themes in Figure 4, which encompassed 5 dimensions: environment, sense-making and assessment, message, tone, and other. There were 2 main dimensions relating to the assessment of acceptability of the tweet: message (content) and tone. However, many factors affected this assessment, including consideration of the venue for sharing. There was a substantive amount of social judgment involved, of both the tweet subject and the tweeter. The tweets also provoked introspection and curiosity. One final category was “other,” which included the acknowledgment that more information was needed to make a judgment on the tweet.

**Figure 5:**
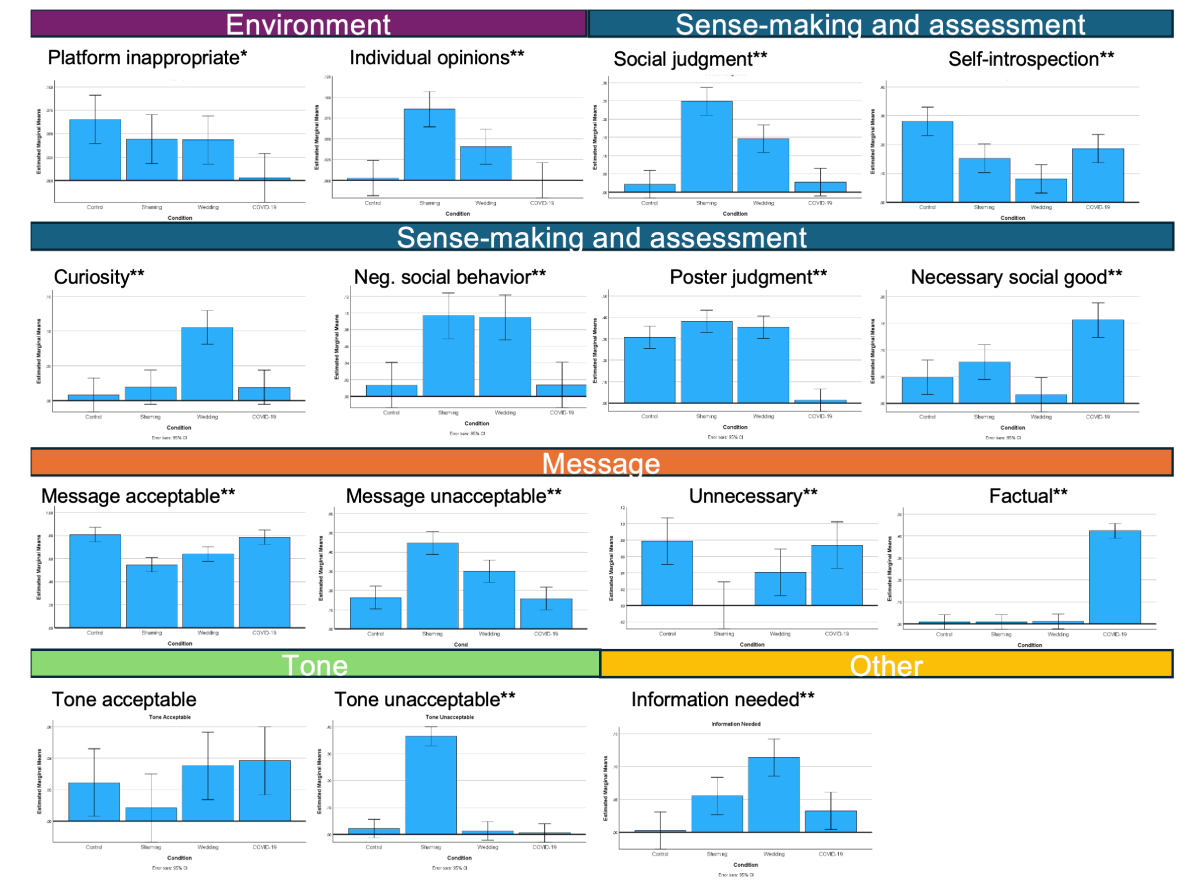
Comparison of participants’ reactions across tweet conditions. Conditions, left to right: control, shaming, wedding, and COVID-19. *P=.01. **P=.001.

When we compare the reactions to the different tweet types on these dimensions, we can unpack factors that explain participants’ reactions to the tweets depending on the type. Overall, the shaming tweet was considered less acceptable in terms of both its message and its tone, and that it represented negative social behavior (Figure 5). With respect to messages being unacceptable, study participants also considered whether tweets were appropriate for the venue and contributed something useful, with the control tweet being seen most often as unnecessary.

There was social judgment involved in the shaming and wedding tweets and of the person who posted the tweet (poster judgment). But there was also the perspective that everyone is entitled to their opinion, and the tweet served a necessary social good. Even in a short message, such as a tweet, participants engaged in sense-making about the poster and the tweet subject, and even positive or neutrally framed posts could evoke judgment (Figure 5; negative social behavior and poster judgment). One striking aspect of the reactions was that the wedding tweet provoked quite a bit of curiosity, reflecting some uncertainty and hesitancy on the part of the participants to pass judgment without knowing more about the situation.

These findings provide insight into why 4 tweets, with their different orientations, might be considered socially acceptable or not. They also explain the increased social media engagement responses for the shaming tweet in that participants’ responses were rooted in distancing the self and in condemning the behavior of the poster.

## Discussion

### Principal Findings

In this study, we conducted an experiment simulating different types of tweets that people might see, one in a shaming context and with 3 other control tweets, and analyzed people’s reactions using a mixed methods approach. Perceived social acceptability and positive emotions generally facilitated engagement, which is consistent with the previous literature observing that positive emotions are more accepted than negative emotions on social media [59], and nationalist appeals evoking hope and enthusiasm resulting in more likes and shares [60].

The effects of negative emotions were more complex, varying with the type of tweet. In addition, in line with previous research reporting that negative emotions, such as fear and worry, increase the likelihood of sharing crisis-related information [61], we observed a greater likelihood to share the COVID-19 pandemic–related tweet in the context of fear. Heightened feelings of shame result in greater commenting on the shaming tweet, suggesting that, when tweet content draws attention to a behavior (eg, lack of adoption of protective measures during the COVID-19 pandemic), a person may feel some sense of culpability, and those who feel more shame may comment or share to divert attention from their possible behaviors or to condemn the behavior of the poster. This is in line with past work reporting that shame may result in the externalization of blame [62]. With respect to anger, when the tweet is aimed at public shaming, increased anger leads to greater engagement across behaviors. According to cognitive appraisal theory [32], anger is an emotion defined by its perception that an external entity is responsible, high perceived personal control, and high certainty of fault [63]. As such, anger results in greater action and decision-making. Thus, if a shaming tweet leads to greater anger, we see participants responding with positive action potential and greater engagement with the tweet.

We found that models that included social acceptability explained more of the variance for liking, whereas the model that included emotion—and the interaction effects involving tweet type—explained more variance for sharing and commenting. This suggests that social acceptability might play a stronger role in explaining why someone might passively engage with a tweet, whereas a cognitive process of interpretation mediated by emotion might explain more active engagement.

By focusing on “visible” traces, the question of how “invisible interactions,” such as choosing to ignore inappropriate content, affect people’s social media use remains unanswered [64]. To answer the question of how the shaming act itself is being interpreted, we performed a qualitative analysis of the open-ended data collected, identifying factors that are important in interpretation and subsequent engagement with online behaviors. Doing so facilitated the identification of salient themes, such as the acceptability of message content and tone, and the suitability of the venue, which may not be apparent in participants’ behaviors. People consider a variety of factors, including but not limited to the platform, their impressions of the poster, themselves, and their reaction situated in time (eg, living in the COVID-19 pandemic time).

Some of the themes identified, such as the importance of the message and tone, are salient in the literature on communication, persuasion, and credibility. For example, message characteristics, source characteristics, emotional valence, and perceived relevance are considered important factors in effect of persuasive messaging [65,66]. This makes sense, as the act of online public shaming includes elements of persuasion. Key takeaways include the following:

- Social media content can affect the audience, whether or not the audience responds visibly.
- In online ecosystems, communicative acts might be thought of as including at least 3 actors: the audience, the social media author, and the object or subject of their gaze (eg, a person or persons being observed).
- The message audience may be cognizant of gaps in knowledge and consider this in formulating their assessment.

### Considering Implicit Norms and Emotions in the Shaping of Online Environments

Considering the findings holistically, we can see that people may respond in diverse ways to the same online content based on their differing interpretations, and that their responses are also temporally dependent. In situations in which people feel threatened, there may be greater acceptance of what otherwise might be considered inappropriate behavior for the greater social good. Moreover, the idea that everyone is entitled to their opinion is another perspective that perhaps creates an online atmosphere in which behavior that may otherwise be perceived as socially unacceptable persists. This finding is congruent with the previous literature reporting that young people find shaming on social media acceptable as a deterrent of future occurrences of that behavior [67].

The finding that the shaming tweet was associated with a greater variability in emotion (Figure 3) is an important one to consider in the shaping of online environments. While each tweet is on some level simply a singular tweet, we are confronted constantly in today’s times with instances of virality. When a tweet goes viral is when one notices it, and yet on a day-to-day basis, there could be other tweets that are eliciting a range of reactions from their audiences, some of which could lead to invisible tensions, only to surface later.

Thus, the findings of this study also suggest the need for programs to mitigate the potential negative consequences of online public shaming. To this end, the reactions of participants to the wedding condition provoke thought. In this case, what we observed was that the tweet provoked questions, and people seemed to want to reserve judgment. Perhaps, consistent with a previous finding that respectful language used increases the probability of respectful language received in return in online disagreements [68], providing education in emotion regulation and respectful online communication could mitigate the impact of online shaming behaviors in digital environments. Moreover, given that many people do not engage actively with social media that they are exposed to and yet it prompts them to think, educational programs that promote dialogue about media could both facilitate understanding of mutual perspectives, as well as serve as an opportunity for people to work through any reactions that they may have to the content.

### The Influence of Social Media Platform on Behavior

In this study, participants’ previous social media use was associated with the behaviors that they indicated they would be more likely to take during the study. For example, Twitter is widely recognized in terms of information diffusion and dissemination in various contexts, including political, crisis, and science communication [69,70], and in this study, use of Twitter was associated with a greater likelihood of liking or sharing. Facebook use denoted a greater likelihood of commenting. Facebook, as a platform, implies greater interaction with known people (eg, friends and family). This could help explain why participants are more likely to engage with posts in terms of liking—as a way of showing support for the material and the poster—but also in terms of commenting—as a way of engaging in a discourse to increase social connections. TikTok, a platform that has been portrayed as a “fun” space that promotes self-presentation as the norm [71], users may become accustomed to sharing as a matter of self-presentation, which may translate to other platforms (such as Twitter, which we simulated in this study), as well. Alternatively, it is possible that those who enjoy sharing are more likely to use TikTok.

Pinterest use was a predictor of all social media behavior. This may be for several reasons. Pinterest, as a platform, can be used both for personal reasons, such as saving content to one’s board for oneself, but also for social reasons, such as curating boards and repinning pins from others. This goal of both personal and social engagement could help explain why there could be an increase in sharing (pinning to one’s own board), liking (to either communicate enjoyment to the poster or to curate for their own boards), and commenting (as a means of making social connections). In addition, extant literature performing a statistical analysis of Pinterest reported “use,” “want to,” “need to,” and “look” as key terms—these terms illustrate both the active and consumption nature of the site [72]. Thus, previous use of social media platforms can predict subsequent behaviors on social media platforms more generally.

### Emotions and Mental Health Implications

Past work on the role of social media in mental health has often investigated the relationship between social media use and anxiety and depression. In a systematic review, Lopes and colleagues observed that usually, the longer the time spent, the worse the outcomes, and other factors, such as emotional involvement and active or passive use, also played a role [73]. Yang et al [74] examined the role of social media interaction during the COVID-19 pandemic and the effects on mental health, finding that people who engaged more in online discussion around the COVID-19 pandemic showed lower life satisfaction and greater anxiety. Similarly, those who engaged in judgment on social media experienced lower life satisfaction. This work suggests that how people engage with social media and the emotions that are evoked during the process can have a direct impact on mental health.

In this study, we found that when the wedding tweet evoked greater feelings of shame, the content was more likely to be shared or commented on. While this behavior could be a way of shifting focus away from a potential implication of their behavior, it might also inadvertently lead to greater rumination and judgment, which are negatively associated with mental health. Alternatively, the act of actively engaging with the content could be a form of emotion regulation—where engaging puts distance between their behavior and emotion, a form of cognitive reappraisal [75]. By understanding more about how people are using engagement with social media as a way of coping with the emotion that is evoked by posts could help better explicate emotional coping mechanisms which could ultimately protect overall mental health.

### Limitations and Future Directions

This study had various limitations. Regarding our experimental stimuli, we used a single social media platform, Twitter. As social media platforms have different features and cultures, how participants might react on one platform may not predict how they might react on another. Nevertheless, Twitter, at the time, was a popular social media platform, and other similar platforms exist and are used all over the world. Thus, our findings not only enhance our understanding of interactions on social media but also inform future research on social media interactions.

In this study, we sought to take a first step at addressing a gap in the literature concerning the reactions of the public to online public shaming in online environments by focusing on online behavioral responses. Future work might collect data to understand what the lasting implications are for mental health when confronted with social media about polarized topics. In addition, our research was situated in the context of the COVID-19 pandemic. Although this choice was well-suited to answer our RQs related to shaming social media posts, our empirical findings could benefit from examination and be translatable to different behavioral forms of shaming (eg, body, smoking, environmentally conscious behavior). Last, although there was some diversity in terms of race and ethnicity, overall, the sample was primarily White, non-Hispanic, and middle-aged, and thus may not be representative of how persons of other backgrounds might react when they encounter social media, and further research is needed with other populations.

### Conclusions

We used a mixed methods approach to characterize people’s reactions to public shaming in online environments. To examine how audiences of online public shaming might respond in terms of effects on mental health and well-being, we performed an experiment in which study participants were shown 1 of 4 tweets: a shaming tweet and 3 different types of control tweets. We then constructed regression models to explore factors that predicted people’s social media behaviors with respect to replying or commenting, sharing, and liking posts. Various factors influenced participants’ responses, including demographic characteristics, their social media preferences, their assessment of the social acceptability and appropriateness of tweets, and the emotional response they had to the tweet. Given the significance of the perceived appropriateness of tweets to the likelihood of participants’ subsequent interactions with the tweet, we performed a qualitative analysis to characterize participants’ impressions and then compared the prominent themes across tweet types. Our main contributions are a model of media interpretation and engagement in online environments, a more nuanced understanding of the complex interplay of factors that may influence people’s response to online public shaming, and recommendations for how to mitigate potential negative consequences.

## Appendix

Multimedia Appendix 1 Instruments.

Multimedia Appendix 2 Derivation of composite indicators.

Multimedia Appendix 3 Checklist for Reporting Results of Internet E-Surveys (CHERRIES).

Multimedia Appendix 4 The effect of risk perception on perceived social acceptability.

## Abbreviations

CHERRIES: Checklist for Reporting Results of Internet E-Surveys
LGBTQ: lesbian, gay, bisexual, transgender, queer
PANAS: Positive and Negative Affect Schedule
RQ: research question

## Data Availability

The datasets generated or analyzed during this study are not publicly available due to the terms under which the authors obtained consent from participants.
