# Supplementary material for: Audience Responses to Online Public Shaming in Online Environments: Mixed Methods Study
Source: J Med Internet Res. 2025 Jul 23;27:e67923. doi: 10.2196/67923 (PMC12329387; doi:10.2196/67923)
Supplement: Multimedia Appendix 1 [file jmir_v27i1e67923_app1.docx]

**Multimedia Appendix 1: Instruments**

**Day 1: Pre-Measure**

1. How often do you use the following social media sites?

N/A – I do not use, Occasionally, 1 – 2 times a day, 3 – times a day, 5 – 6 times a day, 7 – 8 times a day, 9+ times a day

1. Twitter
2. Facebook
3. Instagram
4. TikTok
5. Snapchat
6. YouTube
7. Pinterest
8. Reddit
9. Weibo
10. Other (please specify)
11. Which social media messaging apps do you use (choose all that apply)
    1. WhatsApp
    2. Facebook Messenger
    3. WeChat
    4. Line
    5. Viber
    6. KiK
    7. Telegram Messenger
    8. Discord
    9. Slack
    10. Other (please specify)
12. In general, how likely are you to **share/retweet** an online post on social media?
    (1 = Extremely Unlikely – 5 = Extremely Likely)
13. In general, how likely are you **comment/reply** to an online post on social media?
    (1 = Extremely Unlikely – 5 = Extremely Likely)
14. In general, how likely are you to **“like”** an online post on social media?
    (1 = Extremely Unlikely – 5 = Extremely Likely)
15. How risky do you feel it is to be in a public space without a mask?
    (1 = Not at all Risky - 7 = Very Risky)
16. How comfortable do you feel it is to be in a public space without a mask?
    (1 = Not at all Comfortable – 7 = Very Comfortable)
17. How likely will you be to continue wearing a mask in public spaces?
    (1 = Not at all Likely – 7 = Very Likely)
18. Please list any reasons or considerations for why you might not wear or not wear a mask in public. (open-end)
19. What is your age? Enter in years (e.g., 34)
20. What is your gender identity?
    1. Male
    2. Female
    3. Non-Binary
    4. Prefer Not to Answer
    5. Other (please specify)
21. Are you a native English speaker?
    1. Yes
    2. No
22. How long have you lived in the US?
23. What is your racial identity?
    1. Black or African American
    2. White
    3. American Indian or Alaska Native
    4. Asian
    5. Native Hawaiian or Pacific Islander
    6. Prefer to Say
    7. Other (please specify)
24. Are you of Hispanic/Latino/Spanish origin?
    1. Yes
    2. No
    3. Prefer Not to Say
25. Political ideology (1 = Strongly Liberal – 7 = Strongly Conservative)
    1. On the scale, please indicate which best represented your political identity.
    2. How would you describe your views on social issues?
    3. How would you describe your views on economic issues?
26. Which category best describes your political affiliation?
    1. Democrat
    2. Republich
    3. Independent
    4. Green
    5. Other (please specify)

**Day 2: Main Study**

Participants were randomly shown one of four social media posts:

Shaming Tweet:

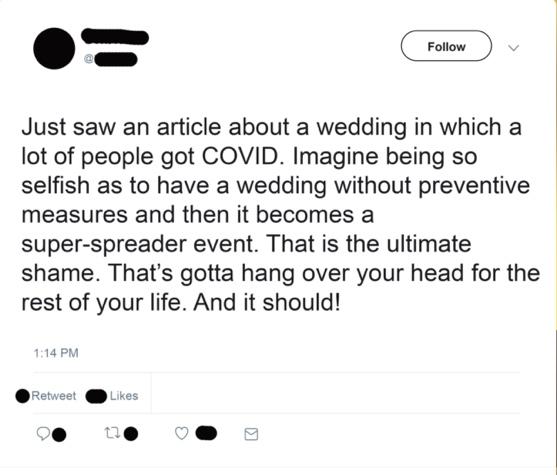


Wedding Tweet:


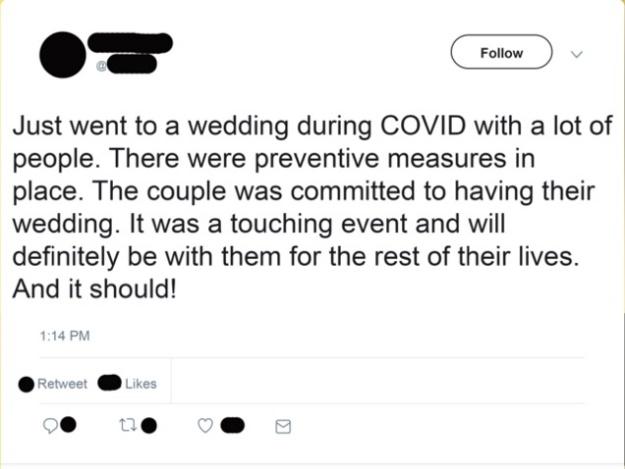


COVID Tweet:


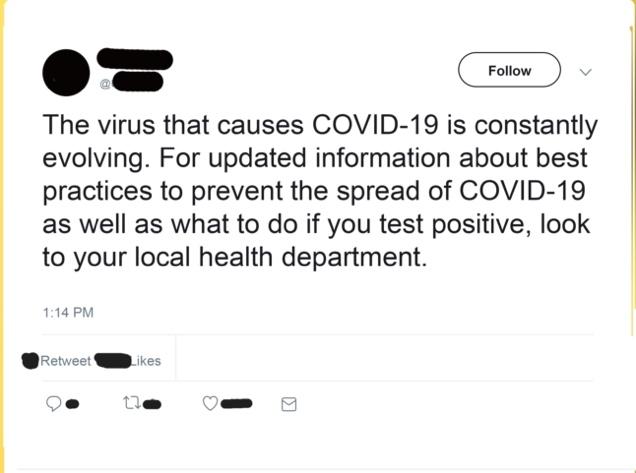


Control Tweet:


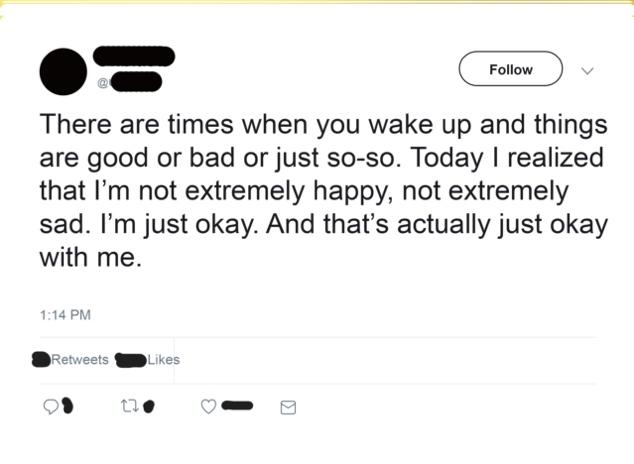


1. Thinking about the post you just saw, what are you thoughts?
2. (PANAS) After reading the post, to what extent do you feel each of the feelings and emotions right now? (1 = Not At all – 9 = A lot)
   1. Interested
   2. Excited
   3. Upset
   4. Scared
   5. Ashamed
   6. Nervous
   7. Attentive
   8. Angry
   9. Happy
   10. Disgusted

1. How likely are you to comment/reply to this online post on social media?
   (1 = Extremely Unlikely – 5 = Extremely Likely)
2. How likely are you to share/retweet this online post on social media?
   (1 = Extremely Unlikely – 5 = Extremely Likely)
3. How likely are you to “like” this online post on social media?
   (1 = Extremely Unlikely – 5 = Extremely Likely)
4. If you were comment on this post on social media, what would you write?
5. How likely are you to look up Covid-19 guidelines?
   (1 = Extremely Unlikely – 5 = Extremely Likely)
6. How socially responsible do you think the post is?
   (1 = Not at all Socially Responsible – 5 = Very Socially Responsible)
7. Please explain why you rated the above question this way.
8. How socially acceptable do you think the post is?
   (1 = Not at all Socially Acceptable – 5 = Very Socially Acceptable)
9. Please explain why you rated the above question this way.
10. How appropriate do you think the post is?
    (1 = Not at all Appropriate – 5 = Very Appropriate)
11. Please explain why you rated the above question this way.
12. What do you think the post was about?
13. How socially responsible do you think the topic of this post is?
    (1 = Not at all Socially Responsible – 5 = Very Socially Responsible)
14. How socially acceptable do you think the topic of this post is?
    (1 = Not at all Socially Acceptable – 5 = Very Socially Acceptable)
15. How appropriate do you think the topic of the post is?
    (1 = Not at all Appropriate – 5 = Very Appropriate)
16. Please explain why you rated the above questions as you did. (open-end)
17. What do you think the goal of this post is?
