# Supplementary material for: Audience Responses to Online Public Shaming in Online Environments: Mixed Methods Study
Source: J Med Internet Res. 2025 Jul 23;27:e67923. doi: 10.2196/67923 (PMC12329387; doi:10.2196/67923)
Supplement: Multimedia Appendix 2 [file jmir_v27i1e67923_app2.docx]

**Multimedia Appendix 2: Derivation of Composite Indicators**

**Composite Emotion Indicators**

Based on cognitive appraisal theory, we expected that the different negative emotions would act differently from each other and from positive emotions. Factor analysis was run to determine whether these theoretical underpinnings of emotion hold true for our sample. Principal component analysis with a varimax rotation solution was used to examine the 10-item PANAS. Initial eigen values indicated that the first two factors explained 39% and 26% of the variance, with the four positive emotions (interested, happy, attentive, and excited; α = .81) loading together and that the six negative emotions (upset, angry, disgusted, scared, ashamed, and nervous, α = .88) loading together. However, looking at the component plot (Figure 2 left), we see that the negative emotions, while uniquely different from positive, do show differences in their spatial components.

Figure 2. Principal Components Analysis of Emotions. Left: Component Plot in Rotated Space (10-item PANAS). Right: Component Plot in Rotated Space for 6-item negative emotion PANAS.


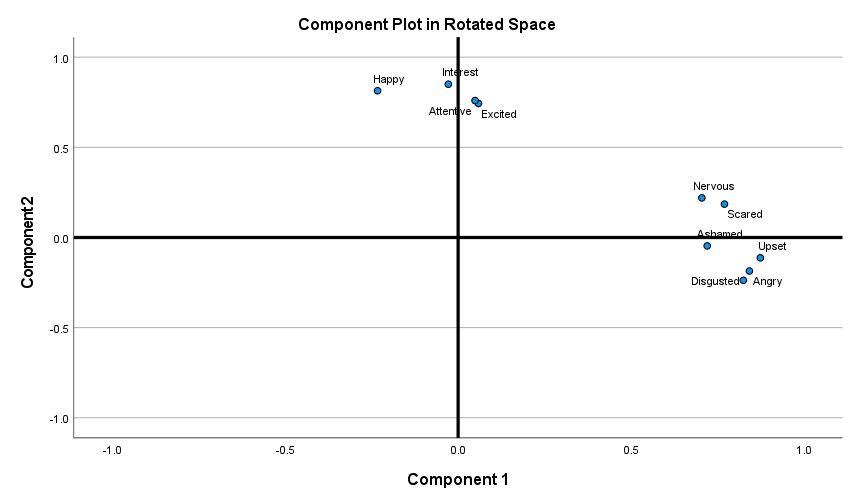

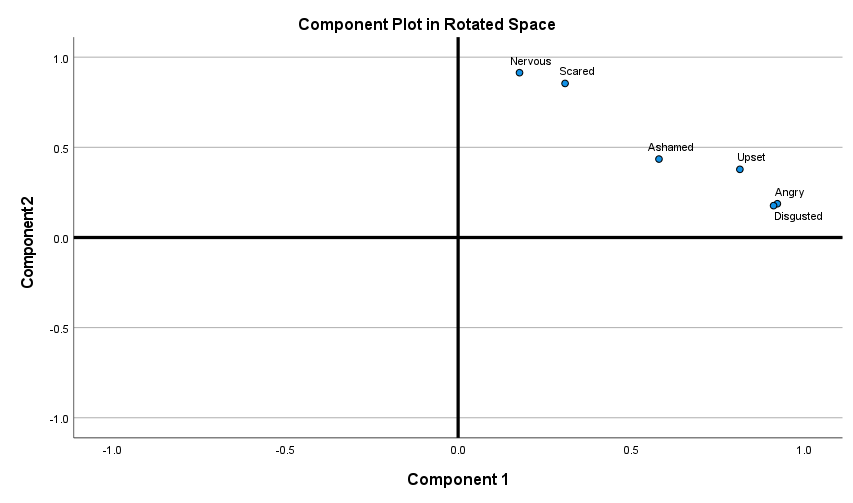


A second principal component factor analysis with varimax rotation was run with the six negative emotions. Results revealed two components, explaining 62.9% and 16.7% of the variance respectively. The component plot (Figure 2 right) supported our theoretic reasoning that fear (e.g., scared and nervous; α = .83) were distinct from anger (e.g., upset, angry, and disgusted; α = .92), while ashamed was distinct from the other two. Thus, we derived composite indicators for three emotion families by averaging items for positive, fear, and anger emotions, and employed shame as a single item.

**Social Acceptability Indicator**

As we are not aware of an appropriate measure to assess social acceptability in this context, we developed our own semantic index by creating three statements which sought to address the concept:

a) How socially responsible do you think the post is?

b) How socially acceptable do you think the post is?

c) How appropriate do you think the post is?

To condense these items, a principal components analysis was run and found that these measures explain 82.5% of the variance. In addition, a reliability analysis found that these items reliably loaded together (α = .89). Thus, we created an index from these items.
