# Supplementary material for: Audience Responses to Online Public Shaming in Online Environments: Mixed Methods Study
Source: J Med Internet Res. 2025 Jul 23;27:e67923. doi: 10.2196/67923 (PMC12329387; doi:10.2196/67923)
Supplement: Multimedia Appendix 4 [file jmir_v27i1e67923_app4.docx]

**Multimedia Appendix 4: The Effect of Risk Perception on Perceived Social Acceptability**

To test whether this individual difference in perceived risk influenced how people engaged with the social media posts, we ran a mediation analysis (Hayes’ PROCESS model 4) [58] to examine whether perceived risk only mediated the relationship between tweet type and social media engagement. We also explored whether perceived risk could moderate perceived social acceptability and whether this could influence social media engagement. A series of moderated mediation models (Hayes’ PROCESS model 7) [58] was run using tweet type as the independent variable, perceived risk as the moderation variable, social acceptability as the mediation variable, and commenting/sharing/liking as the independent variables.

In addition to qualitatively examining individuals’ responses concerning why they rated the tweets to be socially (un)acceptable, given the COVID-19 context, this supplemental analysis explores the effect that one’s perceptions of risk when not wearing a mask in indoor public spaces might have on perceived social acceptability. In part 1 of the study, we asked people about the degree to which they viewed not wearing a mask at an indoor public space were perceived as risky (1 = not at all risky, 7 = very risky). The results of mediation and moderation analyses revealed no effect of perceived risk on commenting, sharing or liking for any of the tweets; however, in certain cases perceived risk affected the perception of social acceptability, which in turn affected subsequent behaviors.

**Commenting:** Analysis revealed no direct effects of tweet type on commenting (all ps >.1), a significant direct effect of social acceptability (b = .2113, t = 5.525, p < .001), a significant index of moderated mediation for control v. shaming tweet (moderated mediation index: b = .0368, .95CI (..0130, .0658)) and a significant moderated mediation for control v. wedding (index: b= -.0533, .95CI (-.0891, -.0241)), but not for control v. COVID (index: b = .0059, .95CI (-.0169, .0296)). This finding suggests that the COVID tweet was rated high on social acceptability regardless of perceived risk; there is no moderation for this condition. However, those who perceived higher risk were more likely to see the shaming post as more acceptable, which would increase commenting, and the wedding post as less acceptable.

**Sharing:** Analysis revealed no direct effects of tweet type on sharing (all ps >.1), a significant direct effect of social acceptability (b = .3841, t = 10.235, p < .001), a significant index of moderated mediation for control v. shaming tweet (moderated mediation index: b = .0670, .95CI (.0258, .1106)) and a significant moderated mediation for control v. wedding (index: b= -.0969, .95CI (-.1443, -.0534)), but not for control v. COVID (index: b = .0106 .95CI (-.0310, .0507)). This finding suggests that the COVID tweet was rated high on social acceptability regardless of perceived risk; there is no moderation for this condition. However, those who perceived higher risk were more likely to see the shaming post as more acceptable, which would increase sharing, and the wedding post as less acceptable.

**Liking:** Analysis revealed no direct effects of tweet type on sharing (all ps >.1), a significant direct effect of social acceptability (b = .8485, t = 19.1322, p < .001), a significant index of moderated mediation for control v. shaming tweet (moderated mediation index: b = .1480, .95CI (.0583, .2360)), and a significant moderated mediation for control v. wedding (index: b= -.2140, .95CI (-.3057, -.1185)) but not for control v. COVID (index: b = .0235 .95CI (-.0662, .1132)). This finding suggests that the COVID tweet was rated high on social acceptability regardless of perceived risk; there is no moderation for this condition. However, those who perceived higher risk were more likely to see the shaming post as more acceptable which would increase liking and the wedding post as less acceptable.

Figure 1. Perceived acceptability as a function of increasing perceived risk of not wearing a mask in indoor public places. Conditions: Blue = Control, Green = Shaming, Red = Wedding, Orange = COVID


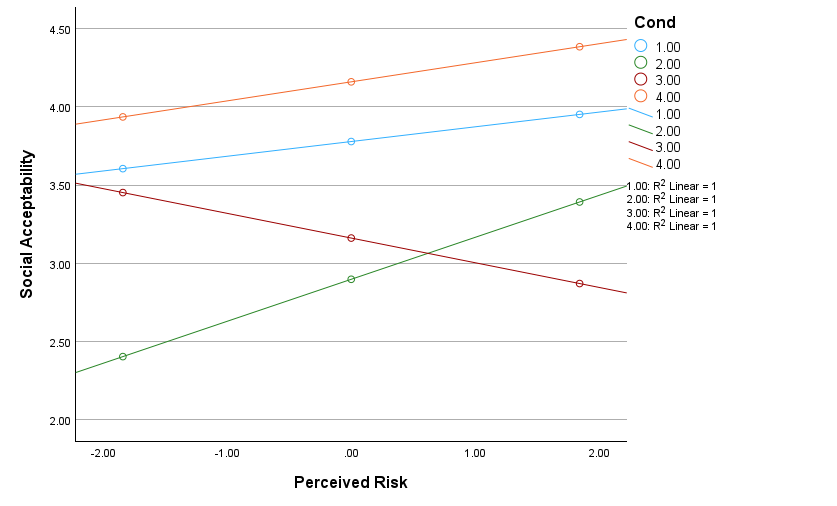


Overall, this additional analysis suggests that there are nuances in what influences perceived social acceptability on social media platforms. In this case, given the public health nature of the posts, the perceived risk of not wearing a mask changed the perception of whether a public shaming post was deemed socially acceptable. This suggests that engaging with public shaming could be a means through which individuals who perceive health behaviors to be crucial to uphold their values and beliefs.
